# Using a RE-AIM framework to identify promising practices in National Diabetes Prevention Program implementation

**DOI:** 10.1186/s13012-019-0928-9

**Published:** 2019-08-14

**Authors:** Kunthea Nhim, Stephanie M. Gruss, Deborah S. Porterfield, Sara Jacobs, Wendi Elkins, Elizabeth T. Luman, Susan Van Aacken, Patricia Schumacher, Ann Albright

**Affiliations:** 1 Division of Diabetes Translation, Centers for Disease Control and Prevention, 4770 Buford Hwy. Mailstop S107-3, Atlanta, GA 30341 USA; 2 RTI International, RTP, NC, and the University of North Carolina School of Medicine, Chapel Hill, NC USA; 3 RTI International, P.O. Box 12194, 3040 E. Cornwallis Road, Rearch Triagle Park, NC 27709-2194 USA

**Keywords:** National Diabetes Prevention Program, Type 2 diabetes prevention, Diabetes prevention program, Diabetes prevention, Lifestyle change intervention, RE-AIM, Implementation evaluation, National organizations, CDC recognition, Diabetes Prevention Recognition Program

## Abstract

**Background:**

The National Diabetes Prevention Program (National DPP) is rapidly expanding in an effort to help those at high risk of type 2 diabetes prevent or delay the disease. In 2012, the Centers for Disease Control and Prevention funded six national organizations to scale and sustain multistate delivery of the National DPP lifestyle change intervention (LCI). This study aims to describe reach, adoption, and maintenance during the 4-year funding period and to assess associations between site-level factors and program effectiveness regarding participant attendance and participation duration.

**Methods:**

The Reach, Effectiveness, Adoption, Implementation, and Maintenance (RE-AIM) framework was used to guide the evaluation from October 2012 to September 2016. Multilevel linear regressions were used to examine associations between participant-level demographics and site-level strategies and number of sessions attended, attendance in months 7–12, and duration of participation.

**Results:**

The six funded national organizations increased the number of participating sites from 68 in 2012 to 164 by 2016 across 38 states and enrolled 14,876 eligible participants. By September 2016, coverage for the National DPP LCI was secured for 42 private insurers and 7 public payers. Nearly 200 employers were recruited to offer the LCI on site to their employees. Site-level strategies significantly associated with higher overall attendance, attendance in months 7–12, and longer participation duration included using self-referral or word of mouth as a recruitment strategy, providing non-monetary incentives for participation, and using cultural adaptations to address participants’ needs. Sites receiving referrals from healthcare providers or health systems also had higher attendance in months 7–12 and longer participation duration. At the participant level, better outcomes were achieved among those aged 65+ (vs. 18–44 or 45–64), those who were overweight (vs. obesity), those who were non-Hispanic white (vs. non-Hispanic black or multiracial/other races), and those eligible based on a blood test or history of gestational diabetes mellitus (vs. screening positive on a risk test).

**Conclusions:**

In a time of rapid dissemination of the National DPP LCI the findings of this evaluation can be used to enhance program implementation and translate lessons learned to similar organizations and settings.

**Electronic supplementary material:**

The online version of this article (10.1186/s13012-019-0928-9) contains supplementary material, which is available to authorized users.

## Background

More than 30 million U.S. adults have diabetes [1], with 90–95% being type 2 diabetes [2]. In addition, the Centers for Disease Control and Prevention (CDC) estimates as many as 84.1 million U.S. adults, more than 1 in 3, have prediabetes [1]. According to the American Diabetes Association (ADA), prediabetes is defined as blood glucose levels that are elevated, though not high enough to be diagnosed as type 2 diabetes [3]. Prediabetes can lead to type 2 diabetes, heart disease, and stroke; however, it is reversible. Multiple studies have demonstrated that lifestyle change programs are effective in preventing or delaying the onset of type 2 diabetes [4–9].

The Diabetes Prevention Program (DPP) randomized clinical trial, initiated in 1996, found that a yearlong intensive lifestyle intervention aimed at reducing risk through healthy eating, physical activity, and stress reduction helped participants lose 5–7% of their body weight and reduce the risk of developing type 2 diabetes by 58% among high risk adults aged ≥ 25 and 71% for those aged ≥ 60 [10]. Since the DPP research study, many translational studies have shown that the structured lifestyle intervention is effective and feasible in community settings to prevent or delay the incidence of type 2 diabetes among people at high risk [11–18]. However, large-scale implementation in the U.S. had not been attempted. In order to do this, a coordinated national effort was needed to increase the supply of quality programs, demand for the program among people at risk, referrals from healthcare providers, and coverage among public and private payers [19].

In response to this need, CDC established the National DPP—a partnership of public and private organizations working to reduce the burden of type 2 diabetes by building the infrastructure for nationwide delivery of the evidence-based lifestyle change intervention (LCI) [19]. The National DPP LCI is a yearlong, structured lifestyle intervention consisting of a minimum of 16 weekly sessions about an hour in length in months 1–6 and a minimum of 6 monthly sessions about an hour in length in months 7–12 [20]. To assure quality and fidelity to scientific evidence, CDC also established the Diabetes Prevention Recognition Program (DPRP), the quality assurance arm of the National DPP. Through the DPRP, CDC recognizes organizations that successfully deliver the yearlong LCI consistent with DPRP Standards and Operating Procedures (Standards) [20]. CDC develops and updates its Standards based on current evidence in the literature, analyses of organizational outcome data, and input from public stakeholders, including organizations delivering the LCI. The DPRP provides technical assistance to delivery organizations and evaluation reports on organizational outcomes linked to the Standards. Additional information about delivery of the National DPP LCI is described elsewhere [20, 21]. CDC grants pending recognition to organizations that have successfully applied to the DPRP, agreeing to use a CDC-approved curriculum and adhere to the specified intensity and duration of the LCI [20]. CDC full recognition is granted to organizations that have implemented the National DPP LCI for at least 12 months and achieved the outcomes described in the Standards [20].

Evaluation of the first 4 years of the program, describing the experience of 14,747 participants, found an average weight loss of 4.2%, with 35.5% of participants achieving ≥ 5% weight loss [21]. In addition, the study found that better retention in the program, with more sessions attended and longer duration from first to last session, resulted in a higher percent of weight loss overall and within subgroups, with a median weight loss of 6% among the 34.6% of participants who attended 17 or more sessions and remained in the program for 7 or more months [21]. Similarly, findings from a 2009 cluster randomized trial of 43 general practices in the United Kingdom (UK) showed that remaining in a type 2 diabetes prevention lifestyle change program was associated with improvements in fasting and 2-h glucose, glycated hemoglobin (HbA1c), weight loss, waist circumference, anxiety, quality of life, and daily step count [22]. A systematic review of translational studies and practical implementation trials of the DPP research study over the last 15 years worldwide also showed that program intensity played a major role in weight loss outcomes [23]. A meta-analysis of randomized clinical trials assessing long-term sustainability of the DPP LCI showed that weight loss was the key factor associated with a delay in type 2 diabetes progression; specifically, every kilogram of weight lost was associated with an additional 7% risk reduction [24]. Despite consistent findings on the effectiveness of the DPP LCI in preventing or delaying onset of type 2 diabetes [10–18, 22–24], there is limited evidence on implementation of the National DPP LCI in real-world settings where people live, work, play, and worship.

In 2012, CDC funded six U.S. national organizations through a 5-year cooperative agreement (*National Diabetes Prevention Program: Preventing Type 2 Diabetes Among People at High Risk* (1212)) to scale and sustain multistate networks to deliver the structured, evidence-based LCI consistent with DPRP Standards in communities nationwide. Funding for the cooperative agreement was allocated based on the organizations’ capacity to reach large numbers of people at risk in multiple states, while working to also obtain health benefit coverage by employers and insurers [25]. The funded national organizations were the American Association of Diabetes Educators, America’s Health Insurance Plans, the Black Women’s Health Imperative, the National Association of Chronic Disease Directors, Optum HealthCare Solutions, and the YMCA of the U.S.

Funded organizations participated in a CDC-led national evaluation of their program implementation to identify promising practices for scaling and sustaining the National DPP. The evaluation was designed using the Reach, Effectiveness, Adoption, Implementation, and Maintenance (RE-AIM) [26] framework. RE-AIM was originally developed by Glasgow, et al. for consistent reporting of research results and later expanded to literature reviews, with particular attention on how to organize study findings on health promotion and disease management in different settings [27, 28]. The RE-AIM framework has been widely used to help guide the translation of research into practice and to help understand the efficacy and effectiveness of programs implemented in real-world community settings [27, 28]. The detailed definitions and levels of measurement for the RE-AIM dimensions are described in Appendix 1: Table 9. This evaluation included qualitative and quantitative measures pertinent to the five dimensions of RE-AIM to assess program implementation over the 4-year funding period.

The present work describes the results of the evaluation of funded organizations’ collective efforts and subsequent lessons learned in scaling up and sustaining the National DPP. The primary objective of this study is to describe program reach, adoption, and maintenance. This study also aims to assess associations between site-level factors and program effectiveness in terms of three outcomes: overall program attendance, attendance in months 7–12, and duration of participation.

## Methods

### Design and setting of the study

#### Population and data sources

A total of 165 CDC-recognized organizations (i.e., sites) were established by the six funded national organizations across the 4-year implementation period (October 1, 2012–September 30, 2016); one site closed without submitting program data, and 28 sites did not submit data during the study period, leaving 136 sites for evaluation (Appendix 2: Fig. 2). By the end of year 4, 14,876 eligible participants had enrolled and attended at least one session at these funded sites. Participant eligibility was based on either self-report or laboratory data from a recent blood test indicating prediabetes (100–125 mg/dl fasting glucose, 140–199 mg/dl 2-h glucose tolerance test, or 5.7–6.4 HbA1c), a previous diagnosis of gestational diabetes mellitus (GDM), or a positive screen on the CDC prediabetes screening test or the American Diabetes Association (ADA) type 2 diabetes risk test [20]. The number of sites participating in the evaluation at any point in time varied across the 4 years (68–132) for several reasons, including new sites were added each year; some sites became independent and continued to deliver the National DPP LCI without cooperative agreement funding; and some sites stopped delivering the program.

Descriptive analyses include program data from the evaluation (*n* = 164 sites), progress reports (*n* = 6 funded national organizations), and data from participants who attended their first session during the implementation period (14,876 eligible individuals at 136 sites). Multilevel analyses include data from sites that had implemented the National DPP LCI for at least 12 months before the end of the evaluation period (*n* = 132) and from participants who attended their first session no later than 1 year before the end of the evaluation period (*n* = 13,767), to evaluate their performance over the yearlong program.

#### Measures and instrument development

The national evaluation framework and accompanying qualitative and quantitative measures were developed by an advisory group made up of CDC staff and external stakeholders. The RE-AIM framework was used to guide instrument development and subsequent enhancements in years 1 and 2 of the funding period, with input from the national organizations and their associated CDC-recognized organizations. The final instruments for data collection from national organizations (Additional file 1) and affiliate sites (Additional file 2) were approved by the Office of Management and Budget (OMB No. 0920-1090; Exp. Date 12/31/2018) in 2015 [29].

The five dimensions of RE-AIM were operationalized for this evaluation as follows. *Program adoption* was measured as the number of CDC-recognized organizations (i.e., sites) offering the LCI, the number of classes offered, the number of new employers offering the LCI on site, the number of employers offering the program as a covered health or wellness benefit for eligible employees, and the number of employees covered by the benefit. *Implementation* was assessed by key features of program delivery: participant recruitment strategies, adaptations made during delivery to meet specific needs of participants, and incentives or behavioral techniques to engage participants or remove barriers. *Maintenance* was defined as the extent to which programs had potential for sustainability, measured by the number of delivery sites achieving full CDC recognition, the number of sites continuing to deliver the program without cooperative agreement funding, and organizational and financial support or program reimbursement from private or public payers. *Reach* was defined as the absolute number and diversity of individuals participating in the program. Characteristics of participant reach included age, gender, race/ethnicity, body mass index (BMI), and eligibility determination. *Effectiveness* was measured by overall participant attendance (number of sessions attended in months 1–12), participant attendance in months 7–12, and duration of participation (days from first to last session within the 12-month program) among eligible participants attending at least one session at sites that offered the LCI for at least 12 months.

*Participant characteristics* were stratified based on sex (male and female), age category at enrollment (18–44, 45–64, and 65+), race/ethnicity (Hispanic, non-Hispanic white, non-Hispanic black, and other), prediabetes eligibility (based on a blood test, previous diagnosis of GDM, or a risk test only), and baseline BMI category (underweight/normal < 25 kg/m2, overweight 25–29 kg/m2, and obesity ≥ 30 kg/m2). The “other” race/ethnicity category includes multiracial, non-Hispanic Asian, non-Hispanic Native Hawaiian/Pacific Islander, non-Hispanic American Indian or Alaska Native, and not reported.

#### Data collection

Findings presented in this paper are from program evaluation data and progress reports submitted annually by the six funded national organizations and their affiliated CDC-recognized organizations and from corresponding participant-level data submitted by the funded sites as part of the CDC recognition process. Evaluation data were extracted and validated by two independent evaluators (KN and SJ), who also provided evaluation technical assistance on data reporting and submission via webinars and ongoing consultation (Additional file 3). For CDC recognition, participant-level data were checked through a series of validations for inconsistent/incomplete reporting by trained statisticians who assisted the sites in correcting and resubmitting their data (Additional file 4). The six national organizations and their associated CDC-recognized organizations submitted program-level data each year with a 99.3% completion rate for evaluation data, and 100% completion rate for progress reports and participant-level data.

#### Statistical analyses

Descriptive statistics (frequencies, means, and ranges) were used to describe program characteristics and outcomes of interest based on the five dimensions of the RE-AIM evaluation framework. Multilevel mixed-effects linear regression analyses, with robust standard error and accounting for national organization-level clustering using a compound symmetry correlation matrix, were used to examine the association between participant-level demographics and site-level implementation strategies and the three primary outcomes (participant overall attendance, attendance in months 7–12, and duration of participation). Forward selection was used to select parsimonious models. Interactions were also tested, and those that were statistically significant at *p* < 0.05 were included in the final models. The Bayesian Information Criterion was used to assess model fit. All analyses were completed in 2017 using SAS 9.3. ArcGIS 10.5.1 was used to create a map. No individual identifiers were reported to CDC, and this study—a project led by CDC with the Research Triangle Institute (RTI) as its contractor—was determined by CDC’s and RTI’s Institutional Review Boards not to be research involving human subjects.

## Results

The results are presented according to the principles of the RE-AIM framework. We present data on program adoption, implementation, and maintenance as well as information on program reach to participants and program effectiveness regarding site-level factors associated with participant overall attendance, attendance in months 7–12, and duration of participation.

### Program adoption

**Fig. 1:**
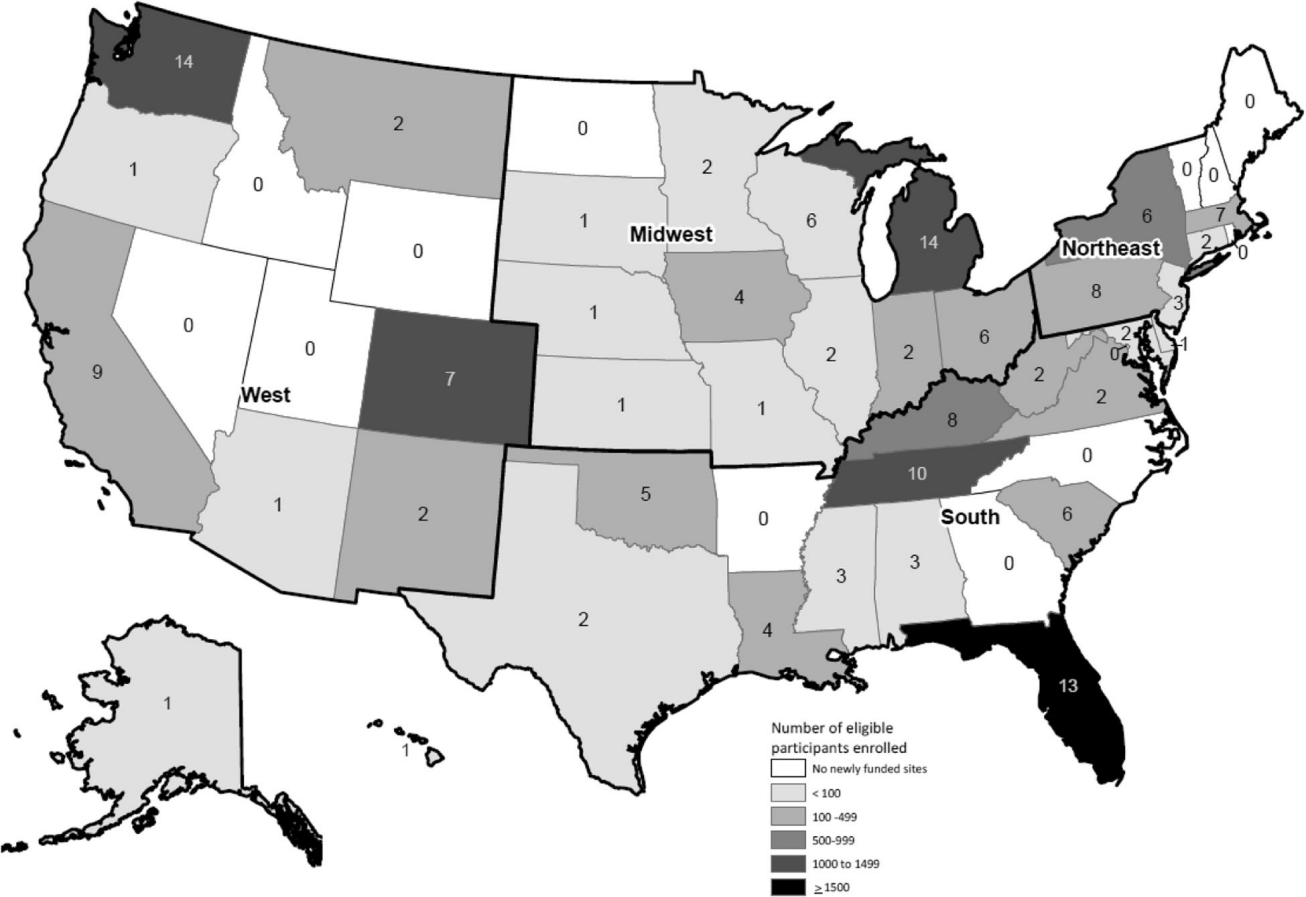
Total number of funded sites^1^ from years 1 to 4 and participants enrolled by state, September 30, 2016: ^1^Funded Sites = CDC-recognized organizations delivering the National Diabetes Prevention Program lifestyle change intervention that were funded through the CDC’s Cooperative Agreement DP12-1212. Note: The number in each state represents number of funded sites; participant eligibility was based on a blood test indicating prediabetes, a diabetes risk test, or a previous diagnosis of gestational diabetes mellitus

**Fig. 2:**
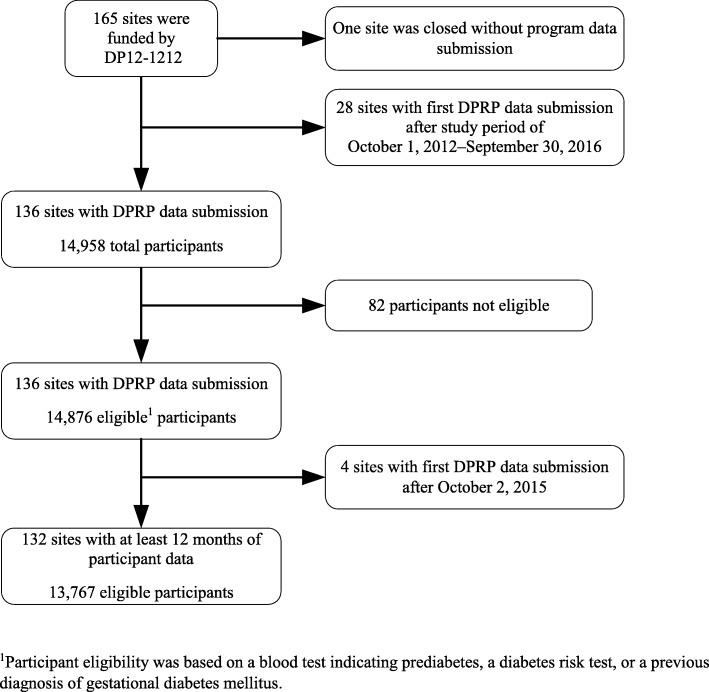
Flow chart for analysis sample. ^1^Participant eligibility was based on a blood test indicating prediabetes, a diabetes risk test, or a previous diagnosis of gestational diabetes mellitus

There was an increase in the number of sites from 68 in year 1 to 132 in year 4, with a total of 164 sites, funded (Table 1). The number of classes offered by these sites increased from 147 in year 1 to 463 in year 4, with a total of 1,239 classes (Table 1). Funded sites were located in 38 states, with the greatest number in Michigan (14), Washington (14), Florida (13), and Tennessee (10); these four states, plus Colorado, also enrolled the highest number of eligible participants per state (≥ 1500) (Fig. 1). By September 2016, the funded sites had enlisted 198 employers to offer the CDC-recognized LCI on site; 27,440 employer groups, including some third-party administrator employer accounts, provided coverage for a total of 5,013,449 employees as a health or wellness benefit (Table 1).

**Table 1:** Characteristics of program adoption among funded sites (*n* = 164)

	Year 1	Year 2	Year 3	Year 4	Total
Number of funded sites per year^1^	68	70	117	132	164^*^
Number of classes offered^2^	147	269	360	463	1239
Number of new employers offering the National DPP lifestyle change intervention (LCI) on site^2^	19	20	117	42	198
Number of new employers offering the National DPP lifestyle change intervention (LCI) as a covered health or wellness benefit for eligible employees^2^	–	–	13,681	13,759	27,440
Number of employees with the National DPP lifestyle change intervention (LCI) as a covered health or wellness benefit^3^	362,733	1,150,562	1,772,529	1,724,925	5,013,449

Funded Sites = CDC-recognized organizations delivering the National Diabetes Prevention Program lifestyle change intervention that were funded through the CDC’s Cooperative Agreement DP12-1212

^1^Source: DP12-1212 program evaluation data, October 1, 2014–September 30, 2016, and DPRP data, October 1, 2012–September 30, 2016

^2^Source: DP12-1212 national organizations’ annual progress reports, September 29, 2012–September 30, 2016

*Total number of sites ever funded by the end of the fourth year funding period. Some sites received funding for only one year, while others for multiple years

–Data not available or not applicable

### Program implementation

Table 2 describes key features of program implementation (recruitment strategies, delivery adaptations, and incentives) among the 164 sites. The majority of sites (90.2%) used a mass media recruitment strategy, such as distributing marketing materials or social media postings. Most (70.1%) reported using a healthcare-based strategy, such as establishing contacts via healthcare provider lists of potential participants or recruiting providers to send postcards/letters during and after patient visits and/or through screenings. Most sites (63.4%) also reported using small media strategies, such as conducting or participating in health fairs or other local events. Less than a third (29.9%) reported participant self-referral or word of mouth as a recruitment strategy.

**Table 2:** Program implementation characteristics among funded sites (*n* = 164)

Implementation strategies	Sites reporting (*n*, %)
Recruitment strategies	*n* = 164^1^
Self-referral or word of mouth	49 (29.9%)
Referral from healthcare providers or systems	115 (70.1%)
Mass media	148 (90.2%)
Small media	104 (63.4%)
Delivery adaptations	*n* = 107^2^
Using cultural themes, images, or sayings; or incorporating cultural dietary restrictions or preferences	40 (37.4%)
Delivering the program bilingually or using a language other than English	22 (20.6%)
Other adaptations	12 (11.2%)
Incentives	*n* = 150^3^
Non-monetary incentive	118 (78.7%)
Monetary incentives	32 (21.3%)
Removal of barriers to access	29 (19.3%)

Source: DP12-1212 program evaluation data, October 1, 2012–September 30, 2016

Funded Sites = CDC-recognized organizations delivering the National Diabetes Prevention Program lifestyle change intervention that were funded through the CDC’s Cooperative Agreement DP12-1212

^1^Question asked in years 1–4

^2^Question asked in years 3 and 4 and for sites existing in the previous year only

^3^Question asked in years 3 and 4 only

Note: Sites implemented more than one strategy per category

Another implementation strategy involved delivery adaptations, in which the sites adjusted program delivery to meet specific needs of participants without deviating from the core components of the LCI and the DPRP Standards. Such adaptations included using cultural themes, images, or sayings, or incorporating cultural dietary restrictions or preferences (37.4% of sites); delivering the program bilingually or in a language other than English (20.6%); or making other adaptations, such as addressing different preferences among male and female participants (11.2%).

Many sites reported using different types of program-funded incentives that were not supported by federal funding, to increase participant enrollment and retention. Three categories of incentives used were non-monetary incentives, such as gym memberships, pedometers, food-measuring devices, or cookbooks (78.7%); monetary incentives, such as coupons or gift cards (value less than $25) (21.3%); and incentives to remove barriers to program access, such as providing child care or transportation passes (19.3%).

### Maintenance

By September 2016, 33 (25%) of the 132 sites with 12 or more months of participant data had achieved full CDC recognition, and 34 sites were no longer funded, 30 of which because they were self-sustaining and no longer needed cooperative agreement funding (Table 3). The number of private insurers and public payers covering the National DPP LCI grew each year. In year 1, nine private insurers and one public payer began offering coverage for their members, employees, and beneficiaries. During years 2 and 3, 26 additional private insurers and six additional public payers (mainly through state or local government employee coverage) added coverage. By the end of the project, a total of 42 private insurers and seven public payers offered coverage for their members, employees, and beneficiaries.

**Table 3:** Characteristics of program maintenance among funded sites (*n* = 164)

	Year 1 (*n* = 68 sites)	Year 2 (*n* = 70 sites)	Year 3 (*n* = 117 sites)	Year 4 (*n* = 132 sites)	Total (*n* = 164 sites)
Number of sites with 12+ months of data	–	68	70	117	132
Number (%) of sites achieving full CDC recognition	–	1 (1.5%)	20 (28.6%)	32 (27.4%)	33 (25.0%)
Number of sites no longer receiving funding	–	0	9	13	34
Number (%) of sites continuing with CDC recognition but no longer receiving DP12-1212 funding	–	0 (0%)	2 (22.2%)	6 (46.2%)	30 (88.2%)
Number of new private insurers covering the National DPP LCI	9	5	21	7	42
Number of new public payers covering the National DPP LCI	1	0	6	0	7

Funded sites = CDC-recognized organizations delivering the National Diabetes Prevention Program lifestyle change intervention that were funded through the CDC’s Cooperative Agreement DP12-1212

Source: DP12-1212 program evaluation data, October 1, 2014–September 30, 2016, and DPRP data, October 1, 2012–September 30, 2016

–Data not available or not applicable

### Reach

The total number of eligible participants across the 4 years of the program was 14,876, with 59.1% eligible based on a blood test or previous diagnosis of GDM and the remaining 40.9% eligible based on the CDC prediabetes screening test or ADA type 2 diabetes risk test (Table 4). The mean age of eligible participants was 56, with an interquartile range of 18. Thirty-nine percent of participants were non-Hispanic white, 12.3% non-Hispanic black, and 11.2% Hispanic, and 37.5% reported other or did not report their race/ethnicity. The majority of participants were female (80.0%), and the majority (63.2%) had obesity, while an additional 29.7% were overweight.

**Table 4:** Reach of the program in funded sites: eligible participant characteristics (*n* = 14,876)

Participant characteristics	Average or number (percentage)
Age (Mean, interquartile range)	56 (47–65)
Race/ethnicity	
Non-Hispanic white	5803 (39.0%)
Non-Hispanic black	1831 (12.3%)
Hispanic	1664 (11.2%)
Other/not reported^1^	5578 (37.5%)
Sex	
Male	2968 (20.0%)
Female	11,904 (80.0%)
Prediabetes eligibility determination	
Blood test or history of GDM	8797 (59.1%)
Prediabetes risk test	6079 (40.9%)
Body mass index (BMI)	
Underweight/normal*	1024 (7.1%)
Overweight	4281 (29.7%)
Obesity	9103 (63.2%)

Source: DPRP data include participants who attended their first session between October 1, 2012 and September 30, 2016

Funded sites = CDC-recognized organizations delivering the National Diabetes Prevention Program lifestyle change intervention that were funded through the CDC’s Cooperative Agreement DP12-1212

Eligible based on a blood test indicating prediabetes, diabetes risk test, or previous diagnosis of gestational diabetes

^1^Other includes multiracial, non-Hispanic Asian, non-Hispanic Native Hawaiian/Pacific Islander, non-Hispanic American Indian or Alaska Native, or not reported

* The data were analyzed in 2017 under the 2015 DPRP Standards that included eligible participants with a BMI ≥ 24 kg/m^2^ (≥ 22 kg/m^2^, if Asian)

### Effectiveness

Results from multilevel analyses showed that sites using self-referral or word of mouth as a recruitment strategy were more likely to have higher average participant attendance (increased by 1.4 sessions), attendance in months 7–12 (increased by 0.6 session), and duration of participation (increased by 23 days) over the yearlong LCI, compared with sites that did not use this strategy (Table 5). Sites receiving referrals from healthcare providers/systems had higher participant attendance in months 7–12 (increased by 0.5 session) and longer duration of participation (increased by 34 days) (Table 6). Sites that offered non-monetary incentives to participants (Table 7), and sites adapting program delivery to address participants’ specific cultural needs or preferences (Table 8), had significantly higher overall participant attendance, attendance during months 7–12, and duration of participation, compared with those that did not use such strategies. However, sites using language adaptations, such as delivering the CDC-recognized LCI in a language other than English, or bilingually, had significantly lower overall participant attendance, attendance during months 7–12, and duration of participation, compared with those that did not (Table 8). Nonprofit organizations and insurers tended to have lower participant attendance in months 7–12 and shorter duration of participation, compared with healthcare-based organizations (Tables 7 and 8).

**Table 5:** Impact of self-referral/word of mouth on measures of participant attendance and participation duration [*n* (participants) = 13,352; *n* (sites) = 132]

	Change in number of visits		Duration of participation (days)
	Any participant attendance	Participant attendance in months 7–12	
Site characteristics			
Recruitment strategy: self-referral or word of mouth	1.4*	0.6*	23.2*
Participant characteristics			
Race/ethnicity: Hispanic^1^	− 0.6*	0.1	0.8
Race/ethnicity: non-Hispanic Black^1^	− 0.8*	− 0.2*	− 10.9*
Race/ethnicity: non-Hispanic other^1^	− 0.6*	− 0.1*	− 7.1*
Prediabetes eligibility determination: prediabetes risk test^2^	− 0.7*	− 0.1*	− 11.5*
Age 18–44 years^3^	− 2.8*	− 0.6	− 41.6*
Age 45–64 years^3^	− 1.2*	− 0.2	− 17.8*
Male^4^	0.2	0.0	2.1
BMI: underweight/normal^5^	0.1	0.2	2.8
BMI: overweight^5^	0.3*	0.1*	7.1*

Source: DP12-1212 program evaluation data, October 1, 2014–September 30, 2016; DPRP data includes eligible participants who attended their first session between October 1, 2012, and September 30, 2015

*p < 0.05

^1^Reference group: non-Hispanic white

^2^Reference group: blood test or history of gestational diabetes mellitus

^3^Reference group: 65+ years

^4^Reference group: female

^5^Reference group: had obesity

**Table 6:** Impact of healthcare referral on measures of participant attendance and participation duration [*n* (participants) = 13,352; *n* (sites) = 132]

	Change in number of visits		Duration of participation (days)
	Any participant attendance	Participant attendance in months 7–12	
Site characteristics			
Recruitment strategy: healthcare referral	1.0	0.5*	33.7*
Participant characteristics			
Race/ethnicity: Hispanic^1^	− 0.6*	0.1	0.9
Race/ethnicity: non-Hispanic Black^1^	− 0.8*	− 0.2*	− 10.9*
Race/ethnicity: non-Hispanic other^1^	− 0.6*	− 0.1*	− 7.0*
Prediabetes eligibility determination: prediabetes risk test^2^	− 0.6*	− 0.1*	− 11.5*
Age 18–44 years^3^	− 2.8*	− 0.6*	− 41.6*
Age 45–64 years^3^	− 1.2*	− 0.2*	− 17.8*
Male^4^	0.2	0.0	2.1
BMI: underweight/normal^5^	0.1	0.2	2.8
BMI: overweight^5^	0.3*	0.13*	7.1*

Source: DP12-1212 program evaluation data, October 1, 2014–September 30, 2016; DPRP data includes eligible participants who attended their first session between October 1, 2012 and September 30, 2015

*p < 0.05

^1^Reference group: non-Hispanic white

^2^Reference group: blood test or history of gestational diabetes mellitus

^3^Reference group: 65+ years

^4^Reference group: female

^5^Reference group: had obesity

**Table 7:** Impact of incentives on measures of participant attendance and participation duration [*n* (participants) = 13,352; *n* (sites) = 132]

	Change in number of visits		Duration of participatio*n* (days)
	Any participant attendance	Participant attendance in months 7–12	
Site characteristics			
Incentive: monetary	− 0.0	− 0.2	− 2.6
Incentive: non-monetary	1.8*	0.6*	27.8*
Incentive: removal of program participation barriers	− 1.0	− 0.1	− 8.3
Site description: nonprofit organization^1^	− 1.9*	− 0.9*	− 38.1*
Site description: educational^1^	− 0.1	− 0.3	− 6.1
Site description: governmental^1^	− 0.8	− 0.6	− 34.4
Site description: insurer^1^	− 1.5	− 1.0*	− 57.0*
Site description: business^1^	1.3	0.1	13.0
Participant characteristics			
Race/ethnicity: Hispanic^2^	− 0.6*	0.1	1.2
Race/ethnicity: non-Hispanic Black^2^	− 0.8*	− 0.2*	− 10.5*
Race/ethnicity: non-Hispanic other^2^	− 0.6*	− 0.1*	− 6.5*
Prediabetes eligibility determination: prediabetes risk test^3^	− 0.6*	− 0.1*	− 11.2*
Age 18–44 years^4^	− 2.8*	− 0.6*	− 41.8*
Age 45–64 years^4^	− 1.2*	− 0.2*	− 18.0*
Male^5^	0.2	0.0	2.1
BMI: underweight/normal^6^	0.1	0.2	2.8
BMI: overweight^6^	0.3*	0.1*	7.1*

Source: DP12-1212 program evaluation data, October 1, 2014–September 30, 2016; DPRP data includes eligible participants who attended their first session between October 1, 2012, and September 30, 2015

* *p* < 0.05

^1^Reference group: healthcare-based site

^2^Reference group: non-Hispanic white

^3^Reference group: blood test or history of gestational diabetes mellitus

^4^Reference group: 65+ years

^5^Reference group: female

^6^Reference group: had obesity

**Table 8:** Impact of delivery adaptation on measures of participant attendance and participation duration [*n* (participants) = 13,352; *n* (sites) = 132]

	Change in number of visits		Duration of participation (days)
	Any participant attendance	Participant attendance in months 7–12	
Site characteristics			
Curriculum adaptation: language	− 1.9*	− 0.7*	− 32.4*
Curriculum adaptation: cultural or other	1.4*	0.7*	29.5*
Site description: nonprofit organization^1^	− 1.5*	− 0.7*	− 28.8*
Site description: educational^1^	− 0.5	− 0.2	− 7.2
Site description: governmental^1^	0.0	− 0.3	− 18.1
Site description: insurer^1^	− 1.5	− 0.8	− 53.3*
Site description: business^1^	0.7	− 0.1	3.7
Participant characteristics			
Race/ethnicity: Hispanic^2^	− 1.0*	− 0.0	− 8.8
Race/ethnicity: non-Hispanic Black^2^	− 1.3*	− 0.2*	− 15.0*
Race/ethnicity: non-Hispanic other^2^	− 0.6*	− 0.1	− 7.0*
Prediabetes eligibility determination: prediabetes risk test^3^	− 0.6*	− 0.1*	− 11.3*
Age 18–44 years^4^	− 2.8*	− 0.6*	− 42.1*
Age 45–64 years^4^	− 1.2*	− 0.2*	− 18.0*
Male^5^	0.2	0.1	2.2
BMI: underweight/normal^6^	0.1	0.2	2.6
BMI: overweight^6^	0.3*	0.1*	7.1*
Interaction (site and participant characteristics)			
Cultural or other adaptation*Hispanic	1.0*	0.2	20.9*
Cultural or other adaptation*non-Hispanic Black	1.1*	0.1	11.1
Cultural or other adaptation*non-Hispanic other	− 0.0	− 0.1	0.6

Source: DP12-1212 program evaluation data, October 1, 2014–September 30, 2016; DPRP data includes eligible participants who attended their first session between October 1, 2012 and September 30, 2015

*p < 0.05

^1^Reference group: healthcare-based site

^2^Reference group: non-Hispanic white

^3^Reference group: blood test or history of gestational diabetes mellitus

^4^Reference group: 65+ years

^5^Reference group: female

^6^Reference group: had obesity

At the participant level, those who were younger (vs. age 65+), were not non-Hispanic white, and were eligible based on a risk test (vs. a blood test or previous diagnosis of GDM) tended to have lower attendance and duration (Tables 5, 6, 7, and 8). In addition, participants who were overweight had significantly higher attendance, attendance in months 7-12, and duration of participation, compared with those who had obesity (Tables 5, 6, 7, and 8). There were no significant differences among male and female participants (Tables 5, 6, 7 and 8). However, Hispanic participants who attended the LCI at sites that used cultural adaptations had higher attendance and duration of participation than those at sites that did not use cultural adaptations; non-Hispanic black participants who attended the LCI at sites using cultural adaptations had higher attendance than those at sites that did not (Table 8).

## Discussion

Over the 4-year funding period, the six national organizations established a total of 164 new CDC-recognized organizations across 38 states, providing 1,239 LCI classes and enrolling nearly 15,000 participants. This represented 10% of all sites and 17% of all eligible participants in the DPRP as of September 2016. In addition, the funded organizations worked with a total of 27,440 new employer groups to offer the National DPP LCI as a covered health or wellness benefit, covering a total of 5,013,449 employees, members, or beneficiaries of whom a subset would be eligible for the program; 198 of these employer groups offered the LCI on site.

Our results suggest that program sustainability was high; one quarter of the funded sites achieved full CDC recognition during the study period, and most sites (88.2%) continued to offer the National DPP LCI and maintain their CDC recognition after funding ended. Additionally, the funded national organizations influenced nearly 50 insurers to cover the program. Beyond the nearly 15,000 people directly served by the funded sites, these changes in coverage have the potential to reach many more people with prediabetes or at high risk for type 2 diabetes.

Although participants were racially diverse (at least 24% reported racial or ethnic minority status), the reach into some underserved population groups was limited (e.g., 20.0% male participants). While this study did not address specific recruitment strategies for men, it showed no statistically significant difference in attendance and duration of participation among men and women. This finding is similar to a systematic review and meta-analysis of gender-specific differences in diabetes prevention, which found no significant differences in incidence of type 2 diabetes and weight change between men and women who received lifestyle interventions [30]. Several new pilot studies have been adapted from the DPP research study to increase reach in men. For example, “Power Up for Health”, a program facilitated by male lifestyle coaches only, was implemented at five different recreation centers located in disadvantaged neighborhoods across New York City [31]. The study showed improvement in weight loss, depressive symptoms, healthy eating and exercise, and health status of male participants, although recruitment was still challenging [31]. In focus group interviews, male participants indicated that the all-male aspect of the program and its use of male coaches were main facilitators for participation [32]. The addition of interactive components such as exercise or healthy cooking demonstrations was also recommended [32]. Similarly, the adapted Kerala DPP in India incorporated male peer-leaders and offered sessions in the evening and on weekends, which was shown to enhance male participation [33]. Based on analysis of CDC’s DPRP registry as of May 2019, there was increased participation by men (27.4%) in virtual programs, compared with 19.6% of male participants in in-person programs [34].

To address the gender gap and other priority populations, in 2017 CDC began funding ten national/regional organizations with affiliate program delivery sites in at least three states to start new CDC-recognized organizations in underserved areas through a 5-year cooperative agreement (1705). This new project incorporates lessons learned from the current evaluation to address gaps and aims to identify and evaluate strategies to enroll populations currently under-represented in the program relative to their estimated numbers and disease burden, such as men, Medicare beneficiaries, African-Americans, Asian-Americans, Hispanics, American Indians, Alaska Natives, Pacific Islanders, and people with visual impairment or physical disabilities.

The RE-AIM model provided a solid framework to assess the implementation of this project, and offers a unique contribution to the type 2 diabetes prevention literature. It allowed simultaneous examination of both participant-level outcome data and detailed organizational and site-level data. Although this evaluation focuses on implementation of the National DPP LCI based on the work of six national organizations in the U.S., it provides a practical evaluation framework and pragmatic measures, which can help address gaps in current evaluations of global diabetes prevention interventions [35]. This evaluation is especially timely, given that the Centers for Medicare & Medicaid Services (CMS) began payment for Medicare participants in CDC-recognized organizations participating in the Medicare Diabetes Prevention Program (MDPP) Expanded Model effective April 1, 2018 [36, 37]. The MDPP will scale the program to a high risk population of Medicare beneficiaries with reimbursement directly tied to CDC recognition to assure program quality and effectiveness [37].

Program-funded incentives, non-CDC funded, were frequently used by sites and were effective in increasing program utilization and retaining participants. We also found that non-monetary incentives such as access to physical activity, pedometers, food measuring devices, or cookbooks were significantly associated with better outcomes. Similarly, recent findings from the 2017 “We Can Prevent Diabetes” trial [38], a collaborative approach with primary care clinics and the YMCA aimed at Medicaid beneficiaries, found that both monetary and gift card incentives increased enrollment, attendance, and weight loss among low-income, high-risk participants in a yearlong type 2 diabetes prevention program [38]. It is also important to note that there was mixed evidence in the literature on various types of incentives used to promote public health interventions among different populations. For example, Sen et al. studied the effectiveness of two lottery incentives (expected daily value of $2.80 vs. $1.40) for improving adherence to remote-monitoring regimens among patients with poorly controlled diabetes and found no difference in adherence between the two incentive arms [39]. However, the low incentive arm had better monitoring rates relative to controls and had significantly better efficacy than the higher incentive arm once incentives were removed [39]. A systematic review of impact of financial incentives on the implementation of asthma or diabetes self-management showed mixed results on diabetes control impact, but there was evidence in improved process and health outcomes in asthma control [40]. A randomized controlled trial comparing the effectiveness of individual vs. team-based financial incentives to increase physical activity showed that financial incentives awarded for a combination of individual and team performance were most effective for increasing physical activity [41].

Funded sites frequently implemented cultural adaptations to address participants’ needs or preferences, which were positively associated with participants’ overall attendance, attendance in months 7–12, and duration of participation. This finding is consistent with Chesla et al., who adapted the CDC-approved Group Lifestyle Balance curriculum for Chinese Americans with prediabetes, resulting in greater achievement of the 5% weight loss goal when compared to Chinese Americans receiving a non-adapted curriculum [42]. We did not, however, find improved results among sites that used language adaptations. In contrast, a systematic review of studies on translated versions of the DPP curriculum in various communities found that translation strategies, including use of bilingual study personnel, had positive results [43]. Further, Taylor et al. identified low literacy and language difficulties as barriers to successful delivery of the DPP to vulnerable and disadvantaged adults and found tailored and flexible program design to be facilitators [44]. Our study did not collect information on lifestyle coaches’ bilingual status, and participants’ socioeconomic status may have been correlated with other barriers to accessing the LCI. To further investigate these issues, CDC plans to assess factors related to lifestyle coaches’ qualifications, training, and language use in a rigorous evaluation of the new cooperative agreement, 1705. In addition, CDC added an education level as a required data field in the new 2018 DPRP Standards to better assess participants’ socioeconomic status [20].

While few sites (29.9%) reported using self-referral or word of mouth as a recruitment strategy, those that did found it to be effective in retaining participants. In addition, sites receiving referrals from healthcare providers/systems had higher participant attendance and longer duration of participation. However, detailed information on how sites implemented these referral strategies was not collected in this study. These results support a systematic review from the Community Preventive Services Task Force [45] and subsequent 2015 recommendation from the U.S. Preventive Services Task Force for clinicians to screen and refer patients at risk to intensive behavioral counseling interventions that promote a healthful diet and physical activity [46]. The ADA followed in 2016 with recommendations that patients with prediabetes be referred to an intensive diet and physical activity behavioral counseling program [47, 48].

At the participant-level, those who were younger (18–44 and 45–64) had significantly lower overall attendance, attendance in months 7–12, and duration of participation than those 65 years and older. This finding is consistent with a study of a multisite diabetes prevention translational project among American Indians and Alaska Natives showing that younger participants were at higher risk for both short-term (not completing all 16 weekly sessions) and long-term (loss to follow-up) retention failure [49]. This finding is also consistent with another study of a similar program in the UK, which found that attendance per 100,000 population was significantly higher as age increased [50]. Despite limited studies comparing retention strategies among younger vs. older participants, results from alternative delivery modalities such as mobile phone-based (i.e., mHealth) or other technology-assisted interventions seem encouraging as a means to reach and engage younger adults. For example, a randomized controlled trial was conducted to assess autonomous motivation and healthy behaviors among young adults with prediabetes who previously declined participation in a diabetes prevention program offered at no cost [51]. The study showed that retention was significantly higher among participants who received an app plus a physical activity tracker and wireless enabled digital scale than participants in the other two study arms combined [51]. These findings offer insight on potential approaches to engage more young adults in the National DPP LCI.

Although Hispanics and non-Hispanic blacks had lower overall attendance than non-Hispanic whites, those who attended the LCI at sites that used cultural adaptations actually had higher attendance compared with those who attended at sites that did not use cultural adaptations. This finding is consistent with a study on community-based translation of the DPP’s lifestyle intervention in an underserved Latino population [52]. Ruggiero et al. found statistically significant improvements in anthropometrics and behavioral outcomes as well as consistent participant attendance rates with other community-based lifestyle intervention programs focused on type 2 diabetes prevention [52].

Finally, this study found that participants who were overweight had significantly higher overall attendance, attendance in months 7–12, and duration of participation, compared with those who had obesity. This finding is consistent with a study of an intensive behavioral intervention for weight management, which found that lower baseline BMI was independently associated with higher retention [53]. It is also consistent with the Let's Prevent Diabetes trial, which found that those who attended all sessions had lower baseline BMI than those who did not [22].

There are several limitations of this study. First, program implementation data were self-reported by the funded organizations and their delivery sites; however, data reported were rigorously assessed for data quality and completeness through a series of validation checks (Additional files 3 and 4). Second, program implementation strategies were collected at site-level only, not participant-level. Multilevel statistical modeling at the site and participant-level was used to address intra-class correlation between participants attending the lifestyle change classes at the same sites and clustering between funded national organizations. Third, many sites were no longer funded by the end of year 4, which may limit generalizability of the study. However, the majority (88%) of those sites became self-sustained and continued to offer the program without cooperative agreement funding. Finally, this study is an observational, retrospective evaluation of CDC’s funded organizations. There were challenges in reaching some population groups such as males, African-Americans, Asian-Americans, Hispanics, American Indians, Alaska Natives, Pacific Islanders, and people with disabilities. As a result, these groups have been identified as populations of focus in the current cooperative agreement. The results may or may not be generalizable to all population groups and small or local community-based organizations that may not have strong infrastructure or resources.

Findings from this evaluation can assist those offering or supporting the National DPP LCI in increasing participants’ retention and thereby potentially improving their weight loss outcomes [21–23, 54, 55] and reducing their risk of developing type 2 diabetes [22–24, 54, 55]. CDC has incorporated lessons learned from this evaluation to inform strategies in its new cooperative agreement (1705) and evaluation framework. Lessons learned from this evaluation were also used to develop and share a variety of technical assistance resources through the National DPP Customer Service Center [56] with new, established, and potential program delivery organizations, as well as to inform the implementation of the MDPP Expanded Model.

## Conclusions

Results from program adoption at the institutional level, and reach at the participant level, suggest that the National DPP LCI is feasible to implement broadly. In a time of rapid growth for the National DPP, lessons learned from this evaluation may be translated to similar organizations and settings. Findings may also be useful to enhance program implementation for the more than 1600 CDC-recognized organizations operating in the U.S., including those with plans to participate as MDPP suppliers as well as those currently funded through the 1705 cooperative agreement. Effective strategies for increasing participant enrollment and retention in the yearlong lifestyle change intervention have tremendous potential to help these organizations achieve the outcomes proven to prevent or delay onset of type 2 diabetes in those at highest risk.

### Additional files

Additional file 1: RE-AIM Evaluation Instrument for DP12-1212 Grantees. (XLSX 26 kb)

Additional file 2: RE-AIM Evaluation Instrument for DP12-1212 Affiliate Sites. (XLSX 32 kb)

Additional file 3: DP12-1212 Grantee and Affiliate Site Data Review and Validation Process. (DOCX 26 kb)

Additional file 4: 2015 Diabetes Prevention Recognition Program (DPRP) Standards for Data Collection and Validation Process. (DOCX 34 kb)

## Data Availability

The datasets used and/or analyzed during the current study are available from the corresponding author on reasonable request.

## Appendix 1

**Table 9:** The RE-AIM dimensions: definitions and levels of measurement

RE-AIM dimension	Definition	Level of measurement
Reach	The absolute number, proportion, and representativeness of individuals who are willing to participate in a given initiative	Individual
Effectiveness	The impact of an intervention on important outcomes, including potential negative effects, quality of life, and economic outcomes	Individual
Adoption	The absolute number, proportion, and representativeness of settings and intervention agents (people who deliver the program) who are willing to initiate a program	Staff and Organizational
Implementation	The intervention agents’ fidelity to the various elements of an intervention’s protocol, including consistency of delivery as intended and the time and cost of the intervention	Staff and Organizational
Maintenance	The extent to which a program or policy becomes institutionalized or part of routine organizational practices and policies at the setting-level. At the individual level, maintenance is defined as the long-term effects of a program on outcomes 6 or more months after the most recent intervention contact	Individual and Organizational

See ref. [28]

## Abbreviations

CDC: Centers for Disease Control and Prevention
CMS: Centers for Medicare & Medicaid Services
DPRP: Diabetes Prevention Recognition Program
GDM: Gestational Diabetes Mellitus
LCI: Lifestyle change intervention
MDPP: Medicare Diabetes Prevention Program
National DPP: National Diabetes Prevention Program

## References

[1] Centers for Disease Control and Prevention. National diabetes statistics report, 2017 [https://www.cdc.gov/diabetes/pdfs/data/statistics/national-diabetes-statistics-report.pdf]. Accessed 1 Sept 2018.

[2] Centers for Disease Control and Prevention. Diabetes Report Card, 2014. https://www.cdc.gov/diabetes/pdfs/library/diabetesreportcard2014.pdf.

[3] American Diabetes Association. Diagnosing diabetes and learning about prediabetes. [http://www.diabetes.org/diabetes-basics/diagnosis/]. Accessed 21 May 2019.

[4] Crandall JP, Knowler WC, Kahn SE, Marrero D, Florez JC, Bray GA (2008). The prevention of type 2 diabetes. Nat Clin Pract Endocrinol Metab..

[5] Diabetes Prevention Program Research Group (2012). The 10-year cost-effectiveness of lifestyle intervention or metformin for diabetes prevention: an intent-to-treat analysis of the DPP/DPPOS.[Erratum appears in Diabetes Care. 2013 Dec;36(12):4173-5]. Diabetes Care.

[6] Hoerger TJ, Hicks KA, Sorensen SW, Herman WH, Ratner RE, Ackermann RT (2007). Cost-effectiveness of screening for pre-diabetes among overweight and obese U.S. adults. Diabetes Care..

[7] Lindstrom J, Ilanne-Parikka P, Peltonen M, Aunola S, Eriksson JG, Hemio K (2006). Sustained reduction in the incidence of type 2 diabetes by lifestyle intervention: follow-up of the Finnish Diabetes Prevention Study. Lancet..

[8] Tuomilehto J, Lindstrom J, Eriksson JG, Valle TT, Hamalainen H, Ilanne-Parikka P (2001). Prevention of type 2 diabetes mellitus by changes in lifestyle among subjects with impaired glucose tolerance. N Engl J Med..

[9] Zhuo X, Zhang P, Gregg EW, Barker L, Hoerger TJ, Tony P-C (2012). A nationwide community-based lifestyle program could delay or prevent type 2 diabetes cases and save $5.7 billion in 25 years. Health Aff (Millwood)..

[10] Knowler WC, Barrett-Connor E, Fowler SE, Hamman RF, Lachin JM, Walker EA (2002). Reduction in the incidence of type 2 diabetes with lifestyle intervention or metformin. N Engl J Med..

[11] Ali MK, Echouffo-Tcheugui J, Williamson DF (2012). How effective were lifestyle interventions in real-world settings that were modeled on the Diabetes Prevention Program?. Health Aff (Millwood)..

[12] Fianu A, Bourse L, Naty N, Le Moullec N, Lepage B, Lang T (2016). Long-Term Effectiveness of a lifestyle intervention for the primary prevention of type 2 diabetes in a low socio-economic community--an intervention follow-up study on Reunion Island. PLoS One..

[13] Dunkley AJ, Bodicoat DH, Greaves CJ, Russell C, Yates T, Davies MJ (2014). Diabetes prevention in the real world: effectiveness of pragmatic lifestyle interventions for the prevention of type 2 diabetes and of the impact of adherence to guideline recommendations - a systematic review and meta-analysis. Diabetes Care..

[14] Aras RY (2014). Lifestyle interventions to prevent and control type 2 diabetes. Indian J Public Health Res Dev..

[15] Modesti PA (2016). Lifestyle interventions in preventing new type 2 diabetes in Asian populations. Intern Emerg Med.

[16] Van Name MA, Camp AW, Magenheimer EA, Li F, Dziura JD, Montosa A (2016). Effective translation of an intensive lifestyle intervention for Hispanic women with prediabetes in a community health center setting. Diabetes Care..

[17] Eaglehouse YL, Schafer GL, Arena VC, Kramer MK, Miller RG, Kriska AM (2016). Impact of a community-based lifestyle intervention program on health-related quality of life. Qual Life Res..

[18] Vojta D, Koehler TB, Longjohn M, Lever JA, Caputo NF (2013). A coordinated national model for diabetes prevention: linking health systems to an evidence-based community program. Am J Prev Med..

[19] Albright AL, Gregg EW (2013). Preventing type 2 diabetes in communities across the U.S.: the National Diabetes Prevention Program. Am J Prev Med.

[20] Centers for Disease Control and Prevention, Diabetes Prevention Recognition Program. Standards and operating procedures [https://www.cdc.gov/diabetes/prevention/pdf/dprp-standards.pdf]. Accessed 1 Sept 2018.

[21] Ely EK, Gruss SM, Luman ET, Gregg EW, Ali MK, Nhim K (2017). A national effort to prevent type 2 diabetes: participant-level evaluation of CDC’s National Diabetes Prevention Program. Diabetes Care..

[22] Gray LJ, Yates T, Troughton J, Khunti K, Davies MJ (2016). Engagement, retention, and progression to type 2 diabetes: a retrospective analysis of the cluster-randomised “Let’s Prevent Diabetes” Trial. PLoS Med..

[23] Aziz Z, Absetz P, Oldroyd J, Pronk NP, Oldenburg B (2015). A systematic review of real-world diabetes prevention programs: learnings from the last 15 years. Implement Sci..

[24] Haw JS, Galaviz KI, Straus AN, Kowalski AJ, Magee MJ, Weber MB (2017). Long-term sustainability of diabetes prevention approaches: a systematic review and meta-analysis of randomized clinical trials. JAMA Intern Med..

[25] Centers for Disease Control and Prevention. PPHF 2012 - National Diabetes Prevention Program: preventing type 2 diabetes among people at high risk financed solely by 2012 Prevention and Public Health Funds [https://www.cdc.gov/diabetes/prevention/foa/dpp-foa-faqs.pdf]. Accessed 1 Sept 2018.

[26] Glasgow RE, Vogt TM, Boles SM (1999). Evaluating the public health impact of health promotion interventions: the RE-AIM framework. Am J Public Health..

[27] www.RE-AIM.org. Frequently Asked Questions [http://re-aim.org/about/frequently-asked-questions/]. Accessed 21 Apr 2019.

[28] Glasgow RE, Harden SM, Gaglio B, Rabin B, Smith ML, Porter GC (2019). RE-AIM Planning and evaluation framework: adapting to new science and practice with a 20-year review. Front Public Health..

[29] Office of Information and Regulation Affairs. Office of Management and Budget. Formative and Summative Evaluation of the National Diabetes Prevention Program [https://www.reginfo.gov/public/do/PRAViewICR?ref_nbr=201509-0920-001]. Accessed 21 Apr 2019.

[30] Glechner A, Harreiter J, Gartlehner G, Rohleder S, Kautzky A, Tuomilehto J (2015). Sex-specific differences in diabetes prevention: a systematic review and meta-analysis. Diabetologia..

[31] Walker EA, Weiss L, Gary-Webb TL, Realmuto L, Kamler A, Ravenell J (2018). Power Up for Health: pilot study outcomes of a diabetes prevention program for men from disadvantaged neighborhoods. Am J Mens Health.

[32] Realmuto L, Kamler A, Weiss L, Gary-Webb TL, Hodge ME, Pagan JA (2018). Power Up for Health-participants’ perspectives on an adaptation of the National Diabetes Prevention Program to engage men. Am J Mens Health.

[33] Mathews E, Thomas E, Absetz P, D'Esposito F, Aziz Z, Balachandran S (2018). Cultural adaptation of a peer-led lifestyle intervention program for diabetes prevention in India: the Kerala diabetes prevention program (K-DPP). BMC Public Health..

[34] Centers for Disease Control and Prevention. Diabetes Prevention Recognition Program data dashboard. Accessed 21 May 2019.

[35] Galaviz KI, Weber MB, Straus A, Haw JS, Narayan KMV, Ali MK (2018). Global diabetes prevention interventions: a systematic review and network meta-analysis of the real-world impact on incidence, weight, and glucose. Diabetes Care..

[36] Centers for Medicare & Medicaid Services. Medicare Diabetes Prevention Program (MDPP) Expanded Model [https://innovation.cms.gov/initiatives/medicare-diabetes-prevention-program/]. Accessed 1 Sept 2018.

[37] Centers for Medicare & Medicaid Services. Revisions to payment policies under the Physician Fee Schedule and Other Revisions to Part B for CY 2018; Medicare Shared Savings Program Requirements; and Medicare Diabetes Prevention Program [https://www.gpo.gov/fdsys/pkg/FR-2017-11-15/pdf/2017-23953.pdf]. Accessed 1 Sept 2018. 29231695

[38] Desai J, Taylor G, Vazquez-Benitez G, Vine S, Anderson J, Garrett JE (2017). Financial incentives for diabetes prevention in a Medicaid population: study design and baseline characteristics. Contemp Clin Trials..

[39] Sen AP, Sewell TB, Riley EB, Stearman B, Bellamy SL, Hu MF, Tao Y, Zhu J, Park JD, Loewenstein G (2014). Financial incentives for home-based health monitoring: a randomized controlled trial. J Gen Intern Med.

[40] Jackson T, Shields MD, Heaney LG, Kendall M, Pearce CJ, Hui CY (2017). The impact of financial incentives on the implementation of asthma or diabetes self-management: a systematic review. PLoS One..

[41] Patel MS, Asch DA, Rosin R, Small DS, Bellamy SL, Eberbach K (2016). Individual versus team-based financial incentives to increase physical activity: a randomized, controlled trial. J Gen Intern Med..

[42] Chesla CA, Chun KM, Kwong Y, Gay CL, Chi HL, Gu Y (2016). Cultural adaptation of the group lifestyle balance program for Chinese Americans. Diabetes Educ..

[43] Hall DL, Lattie EG, McCalla JR, Saab PG (2016). Translation of the Diabetes Prevention Program to Ethnic Communities in the United States. J Immigr Minor Health..

[44] Taylor J, Cottrell C, Chatterton H, Hill J, Hughes R, Wohlgemuth C, Holt RI (2013). Identifying risk and preventing progression to type 2 diabetes in vulnerable and disadvantaged adults: a pragmatic review. Diabet Med.

[45] The Community Guide. Diabetes: Combined diet and physical activity promotion programs to prevent type 2 diabetes among people at increased risk [https://www.thecommunityguide.org/findings/diabetes-combined-diet-and-physical-activity-promotion-programs-prevent-type-2-diabetes]. Accessed 1 Sept 2018.

[46] The U.S. Preventive Services Task Force. Final recommendation statement: abnormal blood glucose and type 2 diabetes mellitus: screening. [https://www.uspreventiveservicestaskforce.org/Page/Document/RecommendationStatementFinal/screening-for-abnormal-blood-glucose-and-type-2-diabetes]

[47] American Diabetes Association. Standards of medical care in diabetes—2016. Diabetes Care. 39:1–119. 10.2337/cd21-as01 PMC7839613 33551551

[48] American Diabetes Association (2016). 4. Prevention or delay of type 2 diabetes. Diabetes Care..

[49] Jiang L, Manson SM, Dill EJ, Beals J, Johnson A, Huang H (2015). Participant and site characteristics related to participant retention in a diabetes prevention translational project. Prev Sci..

[50] Barron E, Clark R, Hewings R, Smith J, Valabhji J (2018). Progress of the Healthier You: NHS Diabetes Prevention Programme: referrals, uptake and participant characteristics. Diabet Med.

[51] Griauzde D, Kullgren JT, Liestenfeltz B, Ansari T, Johnson EH, Fedewa A (2019). A mobile phone-based program to promote healthy behaviors among adults with prediabetes who declined participation in free diabetes prevention programs: mixed-methods pilot randomized controlled trial. JMIR Mhealth Uhealth.

[52] Ruggiero L, Oros S, Choi YK (2011). Community-based translation of the diabetes prevention program's lifestyle intervention in an underserved Latino population. Diabetes Educ.

[53] Rothberg AE, McEwen LN, Kraftson AT, Ajluni N, Fowler CE, Miller NM, Zurales KR, Herman WH (2015). Factors associated with participant retention in a clinical, intensive, behavioral weight management program. BMC Obes.

[54] Mudaliar U, Zabetian A, Goodman M, Echouffo-Tcheugui JB, Albright AL, Gregg EW, Ali MK (2016). Cardiometabolic risk factor changes observed in diabetes prevention programs in US Settings: a systematic review and meta-analysis. PLoS Med.

[55] Jackson SL, Long Q, Rhee MK, Olson DE, Tomolo AM, Cunningham SA, Ramakrishnan U, Narayan KM, Phillips LS (2015). Weight loss and incidence of diabetes with the Veterans Health Administration MOVE! lifestyle change programme: an observational study. Lancet Diabet Endocrinol.

[56] Centers for Disease Control and Prevention. National Diabetes Prevention Program Customer Service Center [https://nationaldppcsc.cdc.gov]. Accessed 30 Apr 2019.

