# Supplementary material for: Using a RE-AIM framework to identify promising practices in National Diabetes Prevention Program implementation
Source: Implement Sci. 2019 Aug 14;14:81. doi: 10.1186/s13012-019-0928-9 (PMC6694543; doi:10.1186/s13012-019-0928-9)
Supplement: Supplementary file 3 — DP12-1212 Grantee and Affiliate Site Data Review and Validation Process. (DOCX 26 kb) [file 13012_2019_928_MOESM3_ESM.docx]

**DP12-1212 Grantee and Delivery Site Data Review and Validation Process**

1. **Grantee Level Spreadsheets:**
   1. All questions have an answer selected.
   2. Check the skip patterns to make sure the grantees responded appropriately and consistently.
   3. All “Other” answers include a specified answer in the “Other” correspondent fields.
   4. The number of new sites reported match the number of new delivery site data received.
   5. Check response to “NEWSITE_CODE” to make sure they provide correct DPRP ORGCODE for all new sites recruited in current grant year.
   6. For each type of marketing materials “NUMMKMAT_Type” reported lists number of each types distributed “NUMMKMAT_Num”.
   7. For each insurer type “INS_NEW_Type” reported lists a unique market location “INS_NEW_Num”.
   8. Responses for the following variables in the current grant year will be checked against data provided from previous grant year to confirm responses are within reasonable parameters (i.e. should not increase more than 5 times): NUM_SITE, NUMMKMAT, INS_NEW, TOTTRND, TOTTRND_MASTER, DAYSTRND, DAYSTRND_MASTER, AVCOSTRN, NUMEMPEDEMPLY, NUMEMPLY, NUMWELL, NUMEMCON, NUMHCPRC, NUMINSBK, AVCOSTPAR, ORG_INCENT (If reported they offer incentives, clarify that the incentive is in addition to what the sites did NOT reporting only on what the sites did).
   9. If “ORG_BAR” is selected, make sure there are correspondent “ORGSRAT” reported.
2. **Site Level Spreadsheets:**
3. **For Existing Sites that were funded from previous year:**
   1. Ensure all existing sites from previous year provided data in current funding year by checking against previous data submission.
   2. Ensure existing sites submitted the correct existing site-level data template.
   3. All questions have an answer selected.
   4. Check the skip patterns to make sure the sites responded appropriately and consistently.
   5. All “Other” answers include a specified answer in the “Other” box.
   6. Check response to “SITE_ACTIVE”, if “No” make sure they responded to “CLOSE_WHY” and not filling other questions. If they filled out all other questions, but the “CLOSE_WHY”, need to clarify with the grantee if this site received funding in current grant year.
   7. If sites respond No to “PARTICIP_FUND”, make sure they fill out the database "1212 Funded Participants" and make sure they fill out one participant per tab. Double check “PARTICIP” with DPRP data.
   8. Check to make sure participant fees “PAR_PROGCOST_Num” are reported if site indicates that they charge participants and can report the fee “PAR_PROGCOST”.
   9. Number of classes “PROGPLAC1_Num” offered at location is filled out and each location type “PROGPLAC” has a unique zip code “PROGPLAC1_Zip”. If multiple classes are provided with one zip code or if only one class is provided with multiple zip codes, site will be contacted for clarification.
   10. Salary of lifestyle coaches and program coordinator is reported if delivery sites indicate they are paid and can report the value.
   11. “COACHMET_Y5_Total” must equal “COCHCRED_Total”
   12. If “ORG_BAR” is selected, make sure there are correspondent “ORGSRAT” reported.
   13. Responses for the following variables in the current grant year dataset will be checked against data provided in previous grant year to confirm responses are within reasonable parameters (i.e. should not increase more than 5 times):
       - - 1. PROGPLAC: Confirm total number of classes is reasonable
           2. COACHMET: Ensure payment (e.g., no funding needed: volunteer) matches the type of lifestyle coaches (e.g., volunteer). If more than one source of funding is selected, site must provide all types of funding by number of coaches; ensure “COACHMET_Y5_Total” is comparable to total number of coaches in previous grant year.
           3. LIFESAL_Num, COORDSAL_Num, PAR_PROGCOST_Num: Run distributions of all cost data and find the outliers for clarification with the sites. Check costs against data submitted in previous grant year.
           4. COCHCRED: Ensure only one credential per coach and compare with previous grant year data.
           5. Total number of program coordinators “COORDSAL_Total” must correspond to “COORDSAL” and comparable with Previous grant year data. Otherwise, need to clarify with the sites.
           6. SITE_DESC: Clarify if any responses changed from previous grant year as responses have been pre-populated by CDC.
           7. PAR_NEW: Clarify if sites select “No new participants in current grant year” to make sure they didn’t enroll any new participants in current grant year.
           8. SVULPOP: Clarify with sites if no responses are selected to make sure they know only data captured in the database will be considered a targeted vulnerable population. No inference for targeted vulnerable population from the grantees’ work in previous years.
           9. SITE_INCENT: Validate against previous grant year submission to ensure sites report all incentives offered in current grant year and not only new incentives (different from previous grant year) offered in current grant year.
4. **For New Sites that were funded only in current grant year:**
5. Ensure new sites submitted the correct New Site Database
6. Make sure they provide correct DPRP ORGCODE for all new sites funded in current grant year
7. All questions have an answer selected.
8. Check the skip patterns to make sure the sites responded appropriately and consistently.
9. All “Other” answers include a specified answer in the “Other” box.
10. If sites respond No to “PARTICIP_FUND”, make sure they fill out the database "1212 Funded Participants" and make sure they fill out one participant per tab. Double check “PARTICIP” with DPRP data.
11. If response to “CLASSDAT” is older than 2016, need to clarify with the grantee when they funded enrollment for this site.
12. SVULPOP: Clarify with sites if no responses are selected to make sure they know only data captured in the database will be considered a targeted vulnerable population. No inference for targeted vulnerable population from the grantees’ work in previous years.
13. Check to make sure participant fees “PAR_PROGCOST_Num” are reported if site indicates that they charge participants and can report the fee “PAR_PROGCOST”.
14. Number of classes “PROGPLAC1_Num” offered at location is filled out and each location type “PROGPLAC” has a unique zip code “PROGPLAC1_Zip”. If multiple classes are provided with one zip code or if only one class is provided with multiple zip codes, sites will be contacted for clarification.
15. Salary of lifestyle coaches and program coordinator is reported if delivery sites indicate they are paid and can report the value.
16. Make sure one credential “COCHCRED” per coach reported.
17. COACHMET: Ensure payment (e.g., no funding needed: volunteer) matches the type of lifestyle coaches (e.g., volunteer). If more than one source of funding is selected, site must provide all types of funding by number of coaches.
18. “COACHMET_Total” must equal “COCHCRED_Total”
19. Total number of program coordinators “COORDSAL_Total” must correspond to “COORDSAL”. Otherwise, need to clarify with the sites.
20. If “ORG_BAR” is selected, make sure there are correspondent “ORGSRAT” reported.
21. **Data Cleaning**
    1. Import all Excel files received from the grantees and sites into SAS.
    2. Merge all data sets received to make sure correspondent grantees and sites are linked correctly.
    3. Data quality check by running frequency distributions to check for missing data, wrong entries, outliers for all variables and confirming the number of new sites, sites active, and grant years align with the DP12-1212 tracking spreadsheet.
    4. Once current grant year data is clean, merge current grant year with final combined dataset.
    5. Re-code “other” response options and generate higher order constructs, consistent with previous grant year.
