# Supplementary material for: Using a RE-AIM framework to identify promising practices in National Diabetes Prevention Program implementation
Source: Implement Sci. 2019 Aug 14;14:81. doi: 10.1186/s13012-019-0928-9 (PMC6694543; doi:10.1186/s13012-019-0928-9)
Supplement: Supplementary file 4 — 2015 Diabetes Prevention Recognition Program (DPRP) Standards for Data Collection and Validation Process. (DOCX 34 kb) [file 13012_2019_928_MOESM4_ESM.docx]

2015 Diabetes Prevention Recognition Program (DPRP) Standards for Data Collection and validation

Data collection process requirements

- There will be a user friendly data entry and update methods so that users can enter data once and be able to tag additional specific data. Examples:
  - OrgCode entered once when organization is approved and assigned to a OrgCode.
  - Using OrgCode entered as reference, ParticipantCode entered once during enrollment phase
  - Using ParticipantCode entered as reference, State, Race, Ethnicity, Age, Sex, Height, Pre-Diabetes diagnostic fields entered once during enrollment phase
  - Using ParticipantCode entered as reference, Session Date, Weight, PA entered on the date a session is held
- There will be a user friendly select list for user to pick from, which are to be translated to the corresponding codes on the CSV export procedure. Examples:
  - Ethnicity
    - Hispanic or Latino (1)
    - Not Hispanic or Latino (2)
    - Not Reported (9)
  - Sex
    - Male (1)
    - Female (2)
    - Not Reported (9)
  - Race
    - AIAN
      - Yes (1)
      - No (2)
    - ASIAN
      - Yes (1)
      - No (2)
- There will be an option to automatically generate unique key for participant code for each participant. If organization enters their own participant code, then there should be a validation check to confirm the uniqueness of the each participant code entered, not just within the data template, but also against any previous or other concurrent data templates being utilized in the same reporting period.
- There will be a capability to check and confirm all validation rules during the CSV export procedure, alerting users for any errors or warning encountered as defined in the DPRP evaluation data validation rules table.

The DPRP evaluation data validation rules

| **Validation No.** | **Validation Description** | **Expected Result** | **Action on Error** | **Error Code** |
| --- | --- | --- | --- | --- |
| 1 | Check data source for presence of the 18 required variables: ORGCODE, PARTICIP, STATE, GLUCTEST, GDM, RISKTEST, AGE, ETHNIC, AIAN, ASIAN, BLACK, NHOPI, WHITE, SEX, HEIGHT, DATE, WEIGHT, PA | All 18 variables are confirmed and no additional variables are found. | Generate report detailing fatal error and return to the organization with message that file is unacceptable until error is corrected. | FE1 |
| 2 | Check data source for additional variables. | No additional variables are found on the file. | If required variables are present but additional variables are also present, delete columns containing additional variables. Generate report detailing warning with message that file has been accepted, but that future submissions should only include required variables. | W1 |
| 3 | Check data source for containing Personally Identifiable Information (PII). | No PII data are found on the file. | If variables are present which contain PII, then delete file, Generate report detailing error with message that file has not been accepted and that a file not containing PII should be resubmitted. | FE2 |
| 4 | Check data source for presence of data entries in all data fields. | Data entries are found in all data fields for all 18 required variables. | Generate report detailing error and return to the organization with message that file is unacceptable until error is corrected. | FE3 |
| 5 | Check data source to verify that entries for the variable ORGCODE are the same for each record. | The entries for the variable ORGCODE are the same for each record. | Generate error report detailing error and return to the organization with message that file is unacceptable until error is corrected. | FE11 |
| 6 | Compare data source to database to verify that the entry for ORGCODE on the data source is in the database as an approved organization. | The entry for the variable ORGCODE is listed in the database as an approved organization. | Generate error report detailing error and return to the organization with message that file is unacceptable until error is corrected. | FE12 |
| 6 | Check data source to verify that entries for the variable PARTICIP are alphanumeric in type and have no more than 25 characters. | Entries for the variable PARTICIP have no more than 25 alphanumeric characters. | Generate report detailing error and return to the organization with message that file is unacceptable until error is corrected. | FE4 |
| 7 | Check data source for duplicate combinations of PARTICIP and DATE to determine uniqueness of PARTICIP entries within an organization. | All PARTICIP entries assigned within an organization are unique. | Generate report detailing error and return to the organization with message that file is unacceptable until error is corrected. | FE5  Or  W3 |
| 8 | Check data source to verify that, for all records, entries for the variable STATE are the two letter abbreviation for the U.S. state or territory | All entries for the variable STATE are 2 letters long and one of the state or territory abbreviation | Generate report detailing error and return to the organization with message that file is unacceptable until error is corrected. | FE6 |
| 9 | Check data source to verify that the same entry for the variable STATE is documented for each of a participant’s records. | The entry for the variable STATE is the same for each of a participant’s records. | If entry changes once, generate report detailing warning and send a message to organization that file has been accepted but that a change in variable value was noted.  If entry changes more than once, generate error report detailing error and send message to organization that file has been conditionally accepted due to the presence of inconsistencies in the variable value. An explanation for the inconsistencies will be needed before evaluation of the data can proceed. | W2  E1 |
| 10 | Check data source to verify that, for all records, entries for the variable listed below are the integer values 1 or 2.   - GLUCTEST - GDM - RISKTEST - AIAN - ASIAN - BLACK - NHOPI - WHITE - Sex | All entries for the variable are the integers 1 or 2. | Generate report detailing error and return to the organization with message that file is unacceptable until error is corrected. | FE7 |
| 11 | Check data source to verify that the same entry for the variable listed below is documented for each of a participant’s records.   - GLUCTEST - GDM - RISKTEST - AIAN - ASIAN - BLACK - NHOPI - WHITE - Sex | The entry for the variable is the same for each of a participant’s records. | If entry changes once, generate report detailing warning and send a message to organization that file has been accepted but that a change in variable value was noted.  If entry changes more than once, generate error report detailing error and send message to organization that file has been conditionally accepted due to the presence of inconsistencies in the variable value. An explanation for the inconsistencies will be needed before evaluation of the data can proceed. | W2  E1 |
| 12 | Check data source to verify that, for all records, entries for the variable AGE are integers in the range 18-125. | All entries for the variable AGE are integers in the range 18-125. | Generate error report detailing error and return to the organization with message that file is unacceptable until error is corrected. | FE8 |
| 13 | Check data source to verify that the same entry for the variable AGE is documented for each of a participant’s records. | The entry for the variable AGE is the same for each of a participant’s records. | If entry changes once, generate report detailing warning and send a message to organization that file has been accepted but that a change in variable value was noted.  If entry changes more than once, generate error report detailing error and send message to organization that file has been conditionally accepted due to the presence of inconsistencies in the variable value. An explanation for the inconsistencies will be needed before evaluation of the data can proceed. | W2  E1 |
| 14 | Check data source to verify that, for all records, entries for the variable ETHNIC are the integer values 1, 2 or 9. | All entries for the variable ETHNIC are the integers 1, 2 or 9. | Generate report detailing error and return to the organization with message that file is unacceptable until error is corrected. | FE9 |
| 15 | Check data source to verify that the same entry for the variable ETHNIC is documented for each of a participant’s records. | The entry for the variable ETHNIC is the same for each of a participant’s records. | If entry changes once, generate report detailing warning and send a message to organization that file has been accepted but that a change in variable value was noted.  If entry changes more than once, generate error report detailing error and send message to organization that file has been conditionally accepted due to the presence of inconsistencies in the variable value. An explanation for the inconsistencies will be needed before evaluation of the data can proceed. | W2  E1 |
| 16 | Check data source to verify that, for all records, entries for the variable HEIGHT are integers in the range 30-99. | All entries for the variable HEIGHT are integers in the range 30-99. | Generate error report detailing error and return to the organization with message that file is unacceptable until error is corrected. | FE10 |
| 17 | Check data source to verify that the same entry for the variable HEIGHT is documented for each of a participant’s records. | The entry for the variable HEIGHT is the same for each of a participant’s records. | If entry changes once, generate report detailing warning and send a message to organization that file has been accepted but that a change in variable value was noted.  If entry changes more than once, generate error report detailing error and send message to organization that file has been conditionally accepted due to the presence of inconsistencies in the variable value. An explanation for the inconsistencies will be needed before evaluation of the data can proceed. | W2  E1 |
| 18 | Check data source to verify that entries for the variable DATE are in the format mm/dd/yyyy. | Entries for the variable DATE are in the format mm/dd/yyyy. | Generate report detailing error and return to the organization with message that file is unacceptable until error is corrected. | FE15 |
| 19 | Compare data source to database to verify that the entries for DATE on the data source do not represent dates which occur before the entry for DPRPStartDate recorded in the database. | The entries for the variable DATE on the data source do not represent dates which occur before the recorded DPRPStartDate in the database. | Generate report detailing error and return to the organization with message that file has been conditionally accepted but that an explanation for the dates recorded will be necessary before data can be evaluated. | E2 |
| 20 | Check data source to verify that, for all records, entries for the variable WEIGHT are integers in the range 70-999. | All entries for the variable WEIGHT are integers in the range 70-999. | Generate error report detailing error and return to the organization with message that file is unacceptable until error is corrected. | FE13 |
| 21 | Check data source to verify that a single participant, the entry for weight does not change more than 3% from one session to another, given that no sessions are missed. | For an individual participant within a core group, their weight will not vary more than 3% from one session to the next, given that no sessions are missed. | Generate report detailing warning with message that file has been accepted, but that these records should be checked for accuracy. | W4 |
| 22 | Check data source to verify that, for all records, entries for the variable PA are integers in the range 0-999. | All entries for the variable PA are integers in the range 0-999. | Generate error report detailing error and return to the organization with message that file is unacceptable until error is corrected. | FE14 |

| **Warning Code** | **Message** |
| --- | --- |
| W1 | “File contains additional variables, which have been deleted from the file. Please only include required variables in next submission. File has been accepted.” |
| W2 | “Entry for variable changed values once for at least one participant’s records. File has been accepted.” |
| W3 | “Entries for the variable are not unique within the organization. File has been accepted.” |
| W4 | “Entries for variable change between sessions for at least one participant. Records should be checked for accuracy. File has been accepted.” |
|  |  |
| **Error Code** | **Message** |
| E1 | “Entry for variable changed values more than once for at least one participant’s records. File has been conditionally accepted.” |
| E2 | “Entries for variable DATE are potentially invalid. File has been conditionally accepted.” |
| FE1 | “File is missing one or more of the 18 variables listed in the “Data Dictionary” in the “DPRP Standards. File has not been accepted.” |
| FE2 | “Personally Identifiable Information was found on the file. File has not been accepted.” |
| FE3 | “File is missing entries for one or more data fields. File has not been accepted.” |
| FE4 | “One or more entries for variable are not alphanumeric in type and/or contain > 25 characters. File has not been accepted.” |
| FE5 | “Entries for the variable are not unique within an organization, or a participant took more than single session in a day. File has not been accepted.” |
| FE6 | “Entries for the variable must be the two letter abbreviation for the U.S. state or territory. File has not been accepted.” |
| FE7 | “Entries for the variable must be a 1 or 2. File has not been accepted.” |
| FE8 | “Entries for the variable AGE must be integers in the range 18-125. File has not been accepted.” |
| FE9 | “Entries for the variable ETHNIC must be a 1, 2 or 9. File has not been accepted.” |
| FE10 | “Entries for the variable HEIGHT must be integers in the range 30-99. File has not been accepted.” |
| FE11 | “Entries for the variable ORGCODE must be the same for each record. File has not been accepted.” |
| FE12 | “Entry for the variable ORGCODE does not represent an approved organization. File is not accepted.” |
| FE13 | “Entries for the variable WEIGHT must be integers in the range 70-999. File has not been accepted.” |
| FE14 | “Entries for the variable PA must be integers in the range 0-999. File has not been accepted.” |
| FE15 | “Entries for the variable DATE must be in the format mm/dd/yyyy. File has not been accepted.” |
